# Changes in the Operative Corridor in Oblique Lumbar Interbody Fusion Between Preoperative Magnetic Resonance Imaging and Intraoperative Cone-Beam Computed Tomography Using Morphometric Analysis

**DOI:** 10.7759/cureus.8687

**Published:** 2020-06-18

**Authors:** Aqib Zehri, Hector Soriano-Baron, Keyan A Peterson, Carol Kittel, Patrick A Brown, Wesley Hsu, Matthew Neal, Jonathan L Wilson

**Affiliations:** 1 Neurological Surgery, Wake Forest Baptist Health, Winston-Salem, USA; 2 Neurological Surgery, The Johns Hopkins Hospital, Baltimore, USA; 3 Biostatistics and Data Science, Wake Forest University School of Medicine, Winston-Salem, USA; 4 Radiology and Neurological Surgery, Wake Forest Baptist Health, Winston-Salem, USA; 5 Neurological Surgery, Wake Forest University School of Medicine, Winston-Salem, USA; 6 Neurological Surgery, Mayo Clinic, Scottsdale, USA

**Keywords:** lateral lumbar interbody fusion, anterior-to-psoas spinal fusion, degenerative disc disease

## Abstract

Background

The oblique lumbar interbody fusion or anterior-to-psoas (OLIF/ATP) technique relies on a corridor anterior to the psoas and posterior to the vasculature for lumbar interbody fusion. This is evaluated preoperatively with CT and/or MRI. To date, there have been no studies examining how intraoperative, lateral decubitus positioning may change the dimensions of this corridor when compared to preoperative imaging.

Objective

Our objective was to evaluate changes in the intraoperative corridor in the supine and lateral positions utilizing preoperative and intraoperative imaging.

Methods

We performed a retrospective analysis among patients who have undergone an OLIF/ATP approach at two tertiary care centers from 2016 to 2018 by measuring the distance between the left lateral border of the aorta or iliac vessels and anteromedial border of the psoas muscle from L1-L2 through L4-5 disc spaces. We compared this corridor between supine, preoperative MRI axial and intraoperative CT acquired in the right lateral decubitus position.

Results

Thirty-three patients, 15 of whom were female, were included in our study. The average age of the patients was 65.4 years and the average BMI was 31 kg/m^2^. The results revealed a statistically significant increase (p<.05) in the intraoperative corridor from supine to lateral decubitus positioning at all levels. However, age, BMI, and gender had no statistically significant impact on the preoperative versus intraoperative corridor.

Conclusion

This is the first study to provide objective evidence that lateral decubitus positioning increases the intraoperative corridor for OLIF/ATP. Our study demonstrates that lateral decubitus positioning provides a more favorable corridor for the OLIF/ATP technique from L1-L5 disc levels.

## Introduction

The oblique lumbar interbody fusion or anterior-to-psoas (OLIF/ATP) technique is a minimally invasive alternative fusion technique to treat degenerative lumbar scoliosis and disc disease. First described in 1997, the OLIF/ATP provides access to the disc space from L1-S1 via an anterior oblique retroperitoneal approach between the psoas major and the anterior vessels [1-4]. Intraoperatively, the patient is placed in a lateral decubitus position to allow the surgeon to stand and face the ventral aspect of the patient’s spine and access the disc space from an anterior oblique angle. The OLIF/ATP approach is performed on the left side of the spine to avoid the contralateral venous structures and obstruction by the liver [5]. The OLIF/ATP approach affords certain potential advantages over posterior approaches, such as less muscle dissection, quicker postoperative mobility, and less postoperative pain, decreased length of hospital stay, blood loss, intraoperative complications, and earlier time to fusion [6-7]. It also has advantages over the direct lateral approach or transpsoas approach due to avoidance of psoas manipulation and potential lumbar plexus violation with more consistent access to the L4-L5 disc space, which can be obstructed in a direct lateral approach especially with a high riding iliac crest [5,8]. This approach can allow for access up to three disc spaces through the same incision.

Patient selection relies upon the presence of an intraoperative corridor that allows for sufficient access to the anterolateral aspect of the spine with the least amount of retraction on the psoas major [9]. Adequate corridor size has been loosely defined as 10 mm at the least [7]. Preoperative imaging is obtained with the patient in a supine position, and further imaging is routinely obtained intraoperatively with three-dimensional (3D) reconstruction for surgical navigation while the patient is in a lateral decubitus position on a flat radiolucent table.

Morphometric analysis of this corridor is limited to a few studies. The cadaveric analysis was first performed to define the OLIF/ATP corridor. One limitation of the cadaveric studies is that they are not representative of the dynamic shift in anatomy that potentially occurs during surgery [5]. Other studies have analyzed the oblique corridor to the lumbar spine using MRI analysis on non-surgical patients to compare changes in the corridor based on levels, laterality, sex, and age [7,9,10]. Only one study has compared corridor size between images in the supine and lateral decubitus position, and it found the corridor to decrease with lateral decubitus positioning in a healthy, young population [10]. However, there have been no studies examining how the operative corridor changes between supine and lateral decubitus positioning using intraoperative imaging in surgical candidates.

The oblique corridor spanning L1-L5 is defined by the distance between the left lateral border of the aorta, or more caudally by the iliac artery, and the anteromedial border of the psoas major. The purpose of this anatomical study was to examine how the oblique corridor to the anterolateral lumbar spine from L1-L5 is altered by comparing supine preoperative imaging to intraoperative cone-beam CT scans with patients in the right lateral decubitus position without a table break. L5-S1 disc space was not examined due to the variability of the iliac arteries and the need for coronal slices on imaging to truly define this corridor.

Potential shifts in the location of large vascular structures or the psoas major at these levels with positioning may play a key role in safe patient selection and excellent patient outcomes. A comprehensive understanding of how the operative corridor changes during surgery may alter patient selection based on supine axial imaging.

## Materials and methods

Data collection 

**Figure 1:**
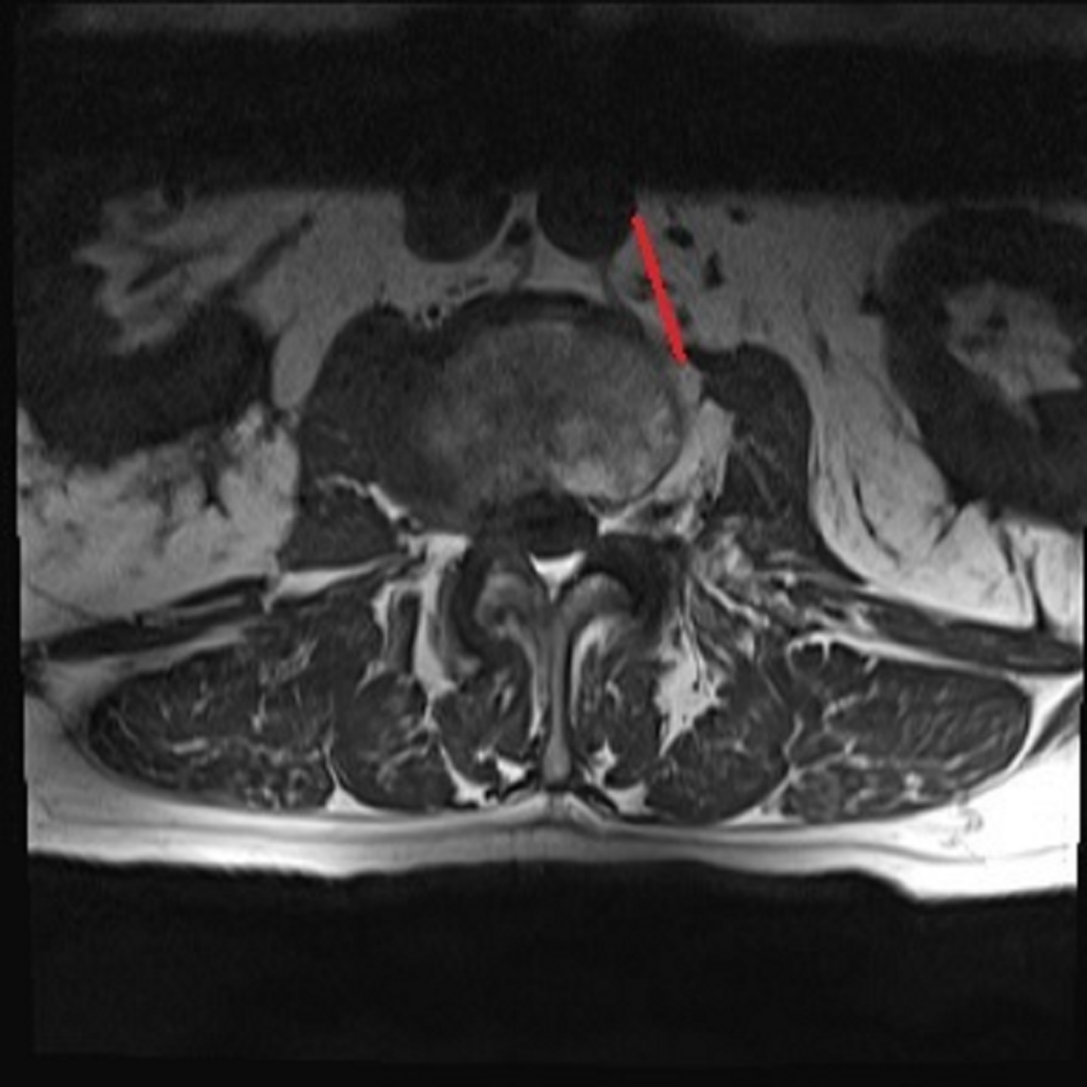
Preoperative corridor measurement using axial MRI T1 weighted image at the L2-L3 disc space as indicated by the red line MRI: magnetic resonance imaging

**Figure 2:**
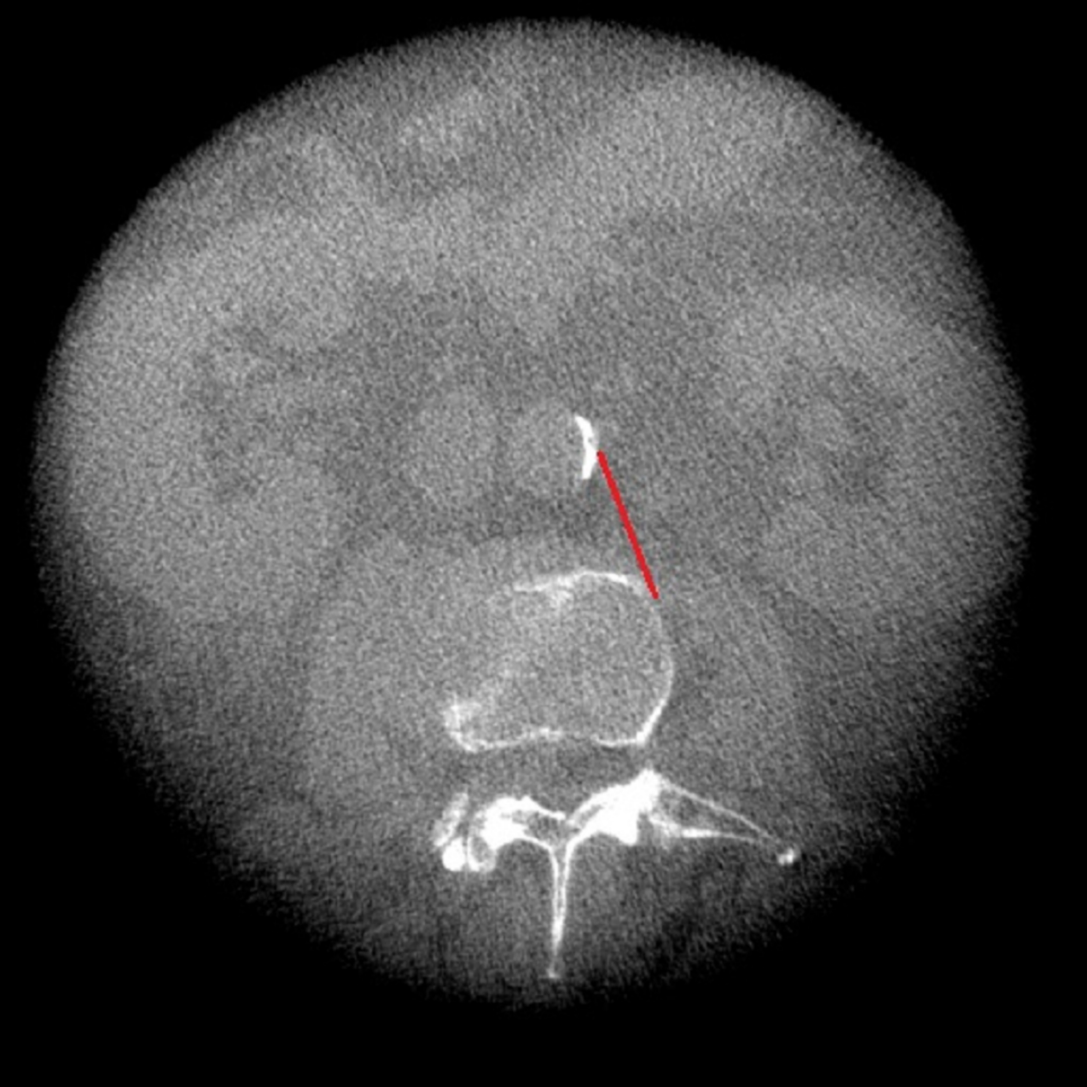
Intraoperative corridor measurement using CT of the same patient as in figure 1 at L2-L3 disc space as indicated by the red line CT: computed tomography

A retrospective chart review was completed to identify all patients of the senior authors; they were individuals of 18-90 years of age, who underwent left-sided OLIF/ATP at two academic medical centers from 2016 to 2018. Patients that were selected for analysis had a preoperative, supine lumbar 1.5T/3T MRI or CT, and intraoperative O-arm imaging in the right lateral decubitus position. Exclusion criteria were as follows: scoliosis curvature greater than 20 degrees (using the Cobb angle from L1-L5), intrinsic abnormalities of the psoas muscle (i.e., tumor, infection, trauma), previous abdominal infections, osseous anomalies of the lumbar spine, or translational anatomy. Once the patients were identified, the charts were reviewed to ensure that inclusion/exclusion criteria were satisfied. Age, gender, BMI, and surgical levels were recorded. For preoperative measurements, the mid-sagittal views were used to identify the disc level on preoperative MRI (T1 weighted image) or CT. The operative corridor between the left lateral border of the aorta or iliac vessels and anteromedial border of the psoas major from L1-2 through L4-5 disc spaces was measured in millimeters on axial images as close to the mid-disk as possible (Figure 1). For intraoperative measurements, lateral X-rays were used to identify lumbar levels since sagittal slices were not obtained. Mid-disc axial CT slices were used to measure the corridor between the left lateral border of the aorta or iliac vessels and anteromedial border of the psoas major from L1-2 through L4-5 disc spaces (Figure 2). The average measurements of the operative corridor were independently obtained by two reviewers, each analyzing images at their respective institutions. Measurements were confirmed independently by a neuroradiologist.

Statistical analysis

All analyses were conducted using R version 3.5.1 (R Foundation for Statistical Computing, Vienna, Austria) and RStudio version 1.1.456 (Integrated Development for R., RStudio, Inc., Boston, MA). Descriptive statistics were calculated such that mean (SD) was used for normally distributed variables and median (range) for nonparametric data. For all analyses, one-tailed hypothesis testing was used with Bonferroni adjusted p-value of <0.0125 interpreted for statistical significance to correct for multiple comparisons at the four measurement levels. The difference in corridor size in the supine and lateral position at each disc level was analyzed using paired sample t-tests. Pearson’s correlation was used to determine the relationship in the average corridor size difference between preoperative versus intraoperative imaging and age or BMI. Wilcoxon rank-sum test was performed to examine whether there was a gender effect on the average corridor size difference between preoperative versus intraoperative corridor size.

## Results

Thirty-three patients, 15 of whom were female, met the inclusion criteria between the two institutions. Two patients were excluded due to a Cobb angle of >20 degrees and one patient had transitional anatomy. Five patients did not have an intraoperative CT obtained during surgery and were excluded. The average age of the participants was 65.4 years, and the average BMI was 31 kg/m^2^. Three patients had a preoperative CT or CT myelogram since an MRI was contraindicated. Eight patients did not have measurements taken at L1-2 and one patient at L3-4 and L4-5 due to poor-quality intraoperative imaging at these levels. After averaging the measures between reviewers, the mean corridor measurements of the corridor at each level on preoperative MRI or CT and intraoperative CT were calculated (Table 1). There was a statistically significant increase (p<.002) in the width of the OLIF/ATP corridor from supine to lateral decubitus positioning at all levels. The greatest average increase in corridor size was noted at L1-2 (3.1 mm) and the least at L4-5 (2.1 mm). When comparing supine preoperative CT to intraoperative CT in three patients, an increase in the corridor size was noted at most levels. There was no statistically significant difference in corridor width between supine and intraoperative lateral decubitus positioning across gender, BMI, or age.

**Table 1:** Average measurement of the operative corridor between preoperative MRI and intraoperative CT and statistical differences MRI: magnetic resonance imaging; CT: computed tomography; SD: standard deviation

	L1-L2	L2-L3	L3-L4	L4-L5
Preoperative MRI, mm, mean (SD)	23.6 (7.5)	21.2 (5.0)	19.5 (5.6)	18.8 (7.3)
Intraoperative CT, mm, mean (SD)	26.1 (7.8)	23.8 (5.6)	22.2 (6.4)	20.8 (7.9)
P-value	0.002	<0.001	<0.001	0.001

## Discussion

Spinal fusion techniques have evolved over the decades, with each offering its own advantages and disadvantages. The first techniques developed were posterior approaches, but these require large incisions, disrupt the posterior ligamentous complex, cause significant postoperative pain, and result in prolonged hospital stays [6]. A transforaminal approach was then developed to allow for a unilateral facetectomy to access the disc space as a more minimally invasive option compared to traditional posterior approaches [11].

Anterior lumbar interbody fusion was then popularized to provide direct access to the L4-5 and L5-S1 levels through a space created by the bifurcation of the great vessel. This approach allows broad exposure to the disc space for larger implant placement and greater spinal deformity correction attributable to required resection of the anterior longitudinal ligament [7]. However, this approach does not allow consistent access to higher lumbar disc spaces due to obstruction by the great vessels and can have complications rates as high as 27% from gastrointestinal, urinary, and vascular injuries even with the assistance of an access surgeon [12].

The lateral transpsoas approach or lateral lumbar interbody fusion was then introduced to overcome the pitfalls of the anterior approach to directly access upper lumbar disc spaces, reduce intraoperative complications, and avoid the need for an access surgeon [13,14]. This approach requires a far lateral incision to enter the retroperitoneal space followed by dissection through the psoas major. This approach affords the advantages of direct access to the anterior disc spaces without mobilizing the great vessels. However, it has a higher incidence of reported lumbar plexus injury and frequently requires neuromonitoring to avoid lumbar plexus damage [14-17]. This approach also does not allow access to the L5-S1 disc space due to obstruction by the iliac crest and vascular limitations [6].

The OLIF/ATP approach helps circumvent some of the limitations related to the approaches previously discussed. Manipulation and dissection of important anatomical structures are limited with the OLIF/ATP approach while providing direct, broad access to the disc space [6,18]. This approach is performed with the patient in a lateral decubitus position with the discectomy and interbody placement completed via an oblique retroperitoneal approach involving blunt dissection through a corridor defined by the anteromedial border of the psoas major and lateral aspect of the aorta or iliac vessels. Tubular dilators are used to keep this corridor open to allow implant placement similar to the lateral transpsoas approach. Patient selection for OLIF/ATP relies on the presence of this corridor on preoperative supine imaging, which has led to studies attempting to define the characteristics of this corridor by morphometric analysis.

The first cadaveric evaluation of this corridor was conducted by Davis et al. in 2014. They demonstrated the presence of an oblique anatomical corridor from L2-S1 in all specimens in a right lateral decubitus position [11]. In this study, the corridor diameters in the static state and with mild psoas retraction, respectively, were as follows: at L2-3: 18.60 and 25.50 mm; at L3-4: 19.25 and 27.05 mm; and at L4-5: 15.00 and 24.45 mm. Wang et al. then published a cadaveric study in 2018 that examined the L1-S1 corridor by comparing a left-sided approach with a right-sided approach [5]. They concluded that the left-sided corridor size was increased at all levels compared to the right-sided corridor width with and without mild psoas retraction.

Three studies have been performed using MRI for morphometric analysis of the OLIF/ATP corridor. Molinares et al. assessed the left-sided corridor from L2-S1 using supine axial MRI. The mean size of the left oblique corridor was 16.04 mm at L2-L3, 14.21 mm at L3-L4, and 10.28 mm at L4-L5, without any difference between genders [9]. Supine axial MRI was utilized by Julian et al. to examine the L1-L5 corridors with a left- versus right-sided comparison similar to the Wang et al. cadaveric study [7]. The mean sizes of the oblique corridor on the left side were greater at the upper lumbar levels in all subjects and greater compared to the right-sided corridor at all levels. Morphometric analysis in a study by Zhang et al. compared the size of the corridor between supine and right lateral decubitus positioning on MRI. They found that the corridor width decreased with positioning, which they attributed to anterior migration of the psoas muscle [10]. They urged caution with this approach based on their concern for decreasing corridor size with lateral positioning. However, this study was done in relatively young, non-obese patients in the Asian population and may not be generalizable to the older, relatively overweight surgical population that may have differing psoas muscle mass.

Our study analyzed the changes in the width of the OLIF/ATP corridor between supine and intraoperative lateral decubitus positioning from L1-L5 in surgical patients. We demonstrated that the size of the corridor via a left-sided approach increases at all levels. The greatest average increase in corridor size was noted at L1-2 (3.1 mm) and the least increase was at L4-5 (2.1 mm). We believe this occurs due to the shift of the great vessels in the retroperitoneal space due to the effects of gravity with lateral positioning with a greater shift from gravity occurring more rostrally. Our study shows findings similar to other morphometric studies using MRI analysis that demonstrated the largest average corridor width at L1-2 and subsequent decrease in corridor width at lower lumbar levels due to the increasing psoas major mass occurring more caudally [7,9,10]. In our study, we found no statistically significant difference in corridor width between supine and intraoperative lateral decubitus positioning across gender, BMI, or age.

There is a possibility that this study overestimates an increase in corridor size given the different imaging modalities utilized for measurements. However, obtaining preoperative supine CT imaging on all surgical patients would expose many patients to unnecessary radiation. The imaging modality variations utilized in this study, while not ideal, most closely resemble those utilized in clinical practice by spine surgeons using intraoperative navigation for the OLIF/ATP approach.

We had presented our preliminary findings on the changes of the OLIF/ATP corridor at the Congress of Neurological Surgeons 2019 Annual Meeting, and subsequently published in the journal Neurosurgery: Zehri AH, Soriano-Baron HE, Hsu W, Neal M, Wilson JL: Changes in the Operative Corridor in Oblique Lumbar Interbody Fusion Between Preoperative Imaging and Intraoperative Cone-Beam Computed Tomography Using Morphometric Analysis; Neurosurgery. 2019, volume: 66. DOI: 10.1093/neuros/nyz310_116.

## Conclusions

We concluded that there is no definite corridor size that ensures the feasibility of the OLIF/ATP technique. Ultimately, many variables influence as to which patients are suitable candidates for this approach. We concur with previous recommendations that a corridor of 10 mm on preoperative imaging should increase the safety and success of the OLIF/ATP approach. Multiple studies have suggested that gentle intermittent retraction on the psoas major will further increase the corridor width by at least 3 mm in addition to the increase of 2-3 mm with lateral positioning noted in the current study. These increases, in addition to a starting 10 mm corridor, should afford a more than adequate corridor to perform this procedure for one- or multi-level spinal stabilization.
